# Continuing Professional Development—Radiation Therapy

**DOI:** 10.1002/jmrs.859

**Published:** 2025-02-10

Maximise your continuing professional development (CPD) by reading the following selected article and answer the five questions. Please remember to self‐claim your CPD and retain your supporting evidence. Answers will be available via the QR code and published in JMRS—Volume 72, Issue 4, December 2025.

### Intrafraction Motion and Impact of Margin Reduction for MR‐Linac Online Adaptive Radiotherapy for Pancreatic Cancer Treatments

A. Fasala, M. Carr, Y. Surjan, P. Daghigh, J. de Leon, A. Burns, V. Batumalai, *Journal of Medical Radiation Sciences* (2025), https://doi.org/10.1002/jmrs.832.

1. What role does abdominal compression play in managing intrafraction motion in pancreatic cancer treatment?
  - It improves patient comfort during radiotherapy
  - It reduces tumour motion by restricting respiratory movement
  - It allows for dose escalation without harming healthy tissue
  - It replaces the need for fiducial markers
2. What was the minimum cine scan duration required for inclusion in the motion analysis?
  - 2 min
  - 4 min
  - 5 min
  - 6 min
3. What specific planning strategy was used in this study for each fraction in stereotactic ablative radiotherapy (SABR) treatment on the MR‐Linac?
  - Deep inspiration breath‐hold
  - Gating strategy
  - Adapt‐to‐shape (ATS) strategy
  - Dose‐painting technique
4. What percentage of the time did the adjusted planning target volume (PTV) remain within the specified imaging range in the study?
  - 78.2%
  - 84.6%
  - 88.2%
  - 92.8%
5. Which outcome aligns with the adjusted planning target volume (PTV) margins?
  - Higher radiation doses to surrounding tissues
  - Improved dose metrics and reduced toxicity risks
  - Increased treatment duration and complexity
  - Reduced need for adaptive radiotherapy

## Answers

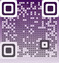

Scan this QR code to find the answers.
